# Herpes simplex virus type 2: Seroprevalence in antenatal women

**DOI:** 10.4103/2589-0557.68994

**Published:** 2010

**Authors:** Shagufta Rathore, Aditi Jamwal, Vipin Gupta

**Affiliations:** Department of Dermatology, Government Medical College and Hospital, Jammu - 180 001, Jammu & Kashmir, India

**Keywords:** Herpes simplex virus type 2, pregnant women, seroprevalence

## Abstract

**Aims:**

To determine the seroprevalence of herpes simplex type 2 (HSV-2) infection in pregnant females, assess the frequency of unrecognized infection and identify the demographic profile and risk factors associated with the seroprevalence.

**Materials and Methods:**

Two hundred randomly selected, asymptomatic pregnant females attending the Obstetrics and Gynecology Outpatient Department for a routine antenatal check-up constituted the study group. Serum specimens were screened for HSV-2 infection by detecting IgG class antibodies against HSV-2-specific glycoprotein G-2 using an enzyme-linked immunosorbent assay kit.

**Results:**

A seroprevalence of 7.5% was found in our study. Seropositivity was maximum in the age group ≥30 years (22.20%), followed by 26–30 years (9.7%), 21–25 years (2.20%) and ≤20 years (0%). HSV-2 seropositivity was found to be significantly associated with increasing age, parity, number of sexual partners, duration of sexual activity and history of abortions (*P* < 0.05). No statistically significant correlation was observed between seropositivity and other demographic variables such as place of residence, education, annual family income and occupation (*P* > 0.05). No statistically significant association of seropositivity with present or past history suggestive of other sexually transmitted infections was found. None of our cases tested positive for human immunodeficiency syndrome (HIV).

**Conclusion:**

A relatively low prevalence of HSV-2 seropositivity was found in our study, with a high frequency of unrecognized and asymptomatic infections. Our findings suggest that type-specific serotesting could be an efficient strategy to diagnose clinically asymptomatic HSV-2 infections and, therefore, to reduce the risk of HSV-2 and HIV sexual transmission by prophylactic counseling against unprotected intercourse. It may also be a useful adjunct in detecting cases who present with symptoms not directly suggestive of genital herpes.

## INTRODUCTION

Schneweis in 1961 demonstrated two serotypes of herpes simplex virus (HSV); herpes simplex virus type 1 (HSV-1) and herpes simplex virus type 2 (HSV-2).[1] HSV-1 and HSV-2 are transmitted by different routes and involve different areas of the body, but there is considerable overlap between epidemiological and clinical manifestations of infections by these viruses. Infection of the genital tract with HSV-1 and HSV-2 can result in genital herpes, but most cases are caused by HSV-2.[2] HSV-2 is primarily transmitted through sexual contact and is one of the most common causes of genital ulcer disease worldwide. The acquisition of HSV-2 infection may be subclinical in some patients so that they are unaware of the disease. About 25% of first symptomatic episode of genital herpes already have antibodies to HSV-2, indicating a past asymptomatic infection.[3]

In pregnancy, primary genital herpes infection manifests clinically in a manner similar to that in a non-pregnant female but with an increased risk of dissemination and mortality.[4] Genital herpes during pregnancy is said to be associated with an increased risk of spontaneous abortions, intrauterine growth retardation (IUGR) and premature delivery.[5]

A dreaded complication of genital herpes in pregnancy is transmission of virus to the newborn, causing neonatal herpes, a disease of high morbidity and mortality. The consequences of neonatal infection are catastrophic; death of the affected neonate or severe neurodevelopmental disability are common. Risk of transmission is 10-times higher in maternal primary infection compared with recurrent infection. Acquisition of infection with seroconversion completed before labor does not appear to affect the outcome of pregnancy, but infection acquired at the time of labor is associated with perinatal morbidity.[5]

HSV infection has been considered to be a risk factor for subsequent or concurrent human immunodeficiency virus (HIV) infection.[6] The increased risk may be a result of discontinuity in the genital mucosal barrier. Herpes lesions are associated with recruitment of CD4 lymphocytes, a factor responsible for increased expression of HIV on the genital mucosa, thereby increasing the viral load and consequent risk of transmission. High titers of HIV virions are found in all genital herpes lesions, which facilitate transmission.[7]

Because HSV causes a lifelong infection with unpredictable reactivation and transmission, detecting antibodies to HSV plays an important role in identifying carriers of this infection. Type-specific tests have been developed that are based on the protein glycoprotein G from HSV-2 (gG2) or glycoprotein (gG1) from HSV 1.[8] Because very limited sequence homology exists between gG1 and gG2, assays based on detecting these type-specific epitopes using either Western blot or enzyme-linked immunosorbent assay (ELISA) can reliably differentiate between antibodies to HSV-1 and antibodies to HSV-2. These tests can also allow in the identification of HSV-2 infection in persons with or without antibodies to HSV-1. Glycoprotein-G-based enzyme immune assays have a sensitivity of 98% and a specificity of 97%.[9,10]

## MATERIALS AND METHODS

The present study was conducted in the outpatient department of Obstetrics and Gynecology, Government Medical College, Jammu, during 2008–2009. The study group comprised of 200 randomly selected asymptomatic pregnant females attending the said hospital for a routine antenatal check-up. Those females presenting with complaints that needed urgent or specialist attention, like hemorrhage, labor pains, severe anemia, jaundice, etc., were excluded from the study. A detailed history was obtained about the demographic details and data relevant to herpes virus infection. After an informed consent, blood samples were collected from the enrolled subjects. Serum was separated and stored at -20°C till conduction of the assay. Serum specimens were screened for HSV-2 infection by detecting IgG class antibodies against HSV-2-specific glycoprotein G-2 using an ELISA kit (RADIM SpA, Italy). The serum specimen was screened for HIV-1 and HIV-2 antibodies by the ELISA technique. Statistical analysis was performed using *t*-test, chi-square test and Fischer test, and referenced for *P*-values for their significance.

## RESULTS

Of the total 200 pregnant females enrolled for the study, 65% belonged to rural and 35% to urban areas. The age of the patients ranged from 16 to 40 years (mean 24.42 ± 4.05). The most common age groups were 21–25 years (44.50%), followed by 26–30 (36.50%), ≥30 (13%) and ≤20 (12%). 65.50% were illiterate. Fifteen cases tested positive for antibodies against HSV-2 and, thus, a seroprevalence of 7.5% was found in our study. The frequency of asymptomatic and unrecognized infections was found to be high; only 6.66% (one out of 15) seropositive cases had a history of genital herpes.

Evaluation of HSV-2 IgG antibodies according to age showed a statistically significant correlation with seropositivity (*P* = 0.012), the risk becoming higher with increasing age. In our study, seropositivity was maximum in the age group ≥30 years (22.20%), followed by 26–30 years (9.70%), 21–25 years (2.20%) and ≤20 years (0%). The various demographic variables studied, like place of residence, annual family income, level of education and occupation (of self and sexual partners) failed to show any significant correlation with seropositivity of HSV-2 (*P* > 0.05) [Table 1].

**Table 1:** Correlation between HSV-2 serology and various sociodemographic factors in antenatal women

Patient characteristic	Total no.(%) of patients	HSV-2 serology		*P*-value
		+ve (%)	-ve (%)	
Age (years)				0.012*
≤20	11 (5.50)	0 (0.00)	11 (100)	
21-25	90 (45.00)	2 (2.20)	88 (97.78)	
26-30	72 (36.00)	7 (9.70)	65 (90.30)	
≥30	27 (13.50)	6 (22.20)	21 (77.80)	
Location				0.41
Rural	130 (65)	10 (7.70)	120 (92.30)	
Urban	70 (35)	5 (7.14)	65 (92.86)	
Occupation				0.54
Unemployed	195 (97.50)	15 (7.70)	180 (92.30)	
Employed	5 (2.5)	0 (0.00)	5 (100)	
Education level				0.56
Illiterate	129 (64.50)	11 (8.53)	118 (91.47)	
Primary and middle	33 (16.50)	2 (6.06)	31 (93.94)	
Sec. and hr. sec.	28 (14.00)	2 (7.14)	26 (92.86)	
Graduate and above	10 (5.00)	0 (0.00)	10 (100)	

* *P*-values ≤0.05 are considered significant

A statistically highly significant association between HSV-2 seropositivity and increasing parity was found (*P* = 0.0001). 1.38% of primiparous females and 25.64% of multiparous females tested positive. A significant correlation between seropositivity to HSV-2 and increasing duration of sexual activity (*P* = 0.003) and multiple sex partners was observed (*P* = 0.018). Only 1% of the cases with ≤ 3 years duration of sexual activity and 22.58% of cases with more than 10 years duration of sexual activity were seropositive. 6.31% of the cases with a single sexual partner and 30% of the cases with multiple sexual partners showed seropositivity to HSV-2. No significant association of seropositivity with early age at first coitus was observed (*P* = 0.123).

Seropositivity was found to be highly associated with history of previous abortions (*P* = 0.001). 19.56% (nine out of 46) of the cases with history of abortions tested positive for antibodies to HSV-2, whereas only 3.89% (six out of 154) cases that had no history of abortion tested positive. No statistically significant association of seropositivity to HSV-2 with present or past history suggestive of other sexually transmitted infections was seen (*P* = 0.08). 12.50% (six out of 48) of the cases had history suggestive of other sexually transmitted infections (STIs) whereas only 5.92% (nine out of 152) who had no such history tested positive [Table 2]. None of our cases tested positive for HIV.

**Table 2:** Correlation between HSV-2 serology and various sexual behavioral markers and risk factors in antenatal women

Patient characteristic	Total no.(%) of patients	HSV-2 serology		*P*-value
		+ve (%)	-ve (%)	
Age at first coitus (years)				0.123
<20	83 (41.50)	10 (12.05)	73 (87.95)	
21-25	106 (53.00)	5 (4.72)	101 (95.28)	
26-30	10 (5.00)	0 (0.00)	10 (100)	
>30	1 (0.50)	0 (0.00)	1 (100)	
Parity				<0.0001*
1	72 (36.00)	1 (1.39)	71 (98.61)	
2	63 (31.50)	1 (1.59)	62 (98.41)	
3	26 (13.00)	3 (11.54)	23 (88.46)	
>4	39 (19.50)	10 (25.64)	29 (74.36)	
No. of sexual partners				0.0187*
Single	190 (95.00)	12 (6.32)	178 (93.68)	
Multiple	10 (5.00)	3 (30.00)	7 (70.00)	
Duration of sexual activity (years)				0.003*
≤3	98 (49)	1 (1.02)	97 (98.98)	
4-7	45 (22.50)	5 (11.11)	40 (88.89)	
7-10	26 (13.00)	2 (7.69)	24 (92.31)	
≥10	31 (15.50)	7 (22.58)	24 (77.42)	
History of abortions				0.001*
Yes	46	9 (19.57)	37 (80.43)	
No	154	6 (3.90)	148 (96.10)	
History suggestive of other STIs				0.08
Yes	48 (24.00)	6 (12.50)	42 (87.50)	
No	152 (76.00)	9 (5.92)	143 (94.08)	

* *P*-values ≤0.05 are considered significant

## DISCUSSION

Epidemiology of genital herpes varies between different countries and between groups of individuals depending on the demographic and clinical characteristics of the population. The seroprevalence of HSV-2 antibodies is an accurate method of determining the epidemiology of this infection. Serological assay utilizing type-specific glycoprotein gG1 and gG2 is more accurate in differentiating between antibodies directed against HSV-1 and HSV-2.

The prevalence of genital herpes has increased markedly in the past few decades. In the present study, a seroprevalence of 7.5% was found among pregnant females. The age range of cases in our study was 16–40 years (mean 24.42 ± 4.05). In contrast to our study, a much higher HSV-2 seroprevalence has been reported from various rural and urban populations from Africa (60–90%[11] and South and North America (30–70%).[12] This could be because of a higher prevalence of promiscuous sexual behavior, large number of sexual partners and high prevalence of other sexually transmitted infections in these communities. Nizami *et al*.[13] reported a seroprevalence of 63.1% in pregnant women whereas Tideman *et al*. reported a seroprevalence of 11.3%[14] and Dan *et al*. reported a seroprevalence of 13.3%.[15] Prevalence in the general population in developing Asian countries appears to be lower (10–30%).[16] Maitra and Gupta[17] found a seroprevalence of 23.3% in a general gynecology clinic and Chawla *et al*.[18] reported a seroprevalence of 7% and 8.6% in two urban communities in Delhi.

In our study, the HSV-2 seroprevalence rose steadily with age (2.2% among women aged 21–25 years to 22.20% among women aged ≥30 years). These findings are comparable to the studies of Breinig *et al*.,[19] Tideman *et al*.[14] and Nizami *et al*.[13] No statistically significant correlation was observed with other demographic variables in our study, such as place of residence, whether rural or urban, education, annual family income, occupation and socioeconomic status. Similar findings were reported by Fleming *et al*.[12] However, Stavraky *et al*.,[20] Breinig *et al*.,[19] Tideman *et al*.[14] and Chawla *et al*.[20] found a significant association between HSV-2 seropositivity and sociodemographic factors while assessing the risk factors for HSV-2 infection in women.

In our study, we also assessed the role of various sexual behavioral markers in the seropositivity to HSV-2 and a significant correlation was observed with increasing number of previous pregnancies, number of sexual partners, duration of sexual activity and history of abortions Breinig *et al*.[19] and Tideman *et al*.[14] reported a significant association between seropositivity and increasing parity. The effect of increasing number of previous pregnancies on seropositivity may not be direct but may be a reflection of increased duration of sexual activity, which itself is a risk factor for HSV seropositivity. Stavraky *et al*.,[20] Cowan *et al*.[9] and Narouz *et al*.[21] observed that patients with multiple sex partners and increasing duration of sex activity and early age of sexual intercourse were at a higher risk of being seropositive to HSV-2. Our study failed to demonstrate an increased risk of seropositivity with early age of first intercourse. Breinig *et al*.[19] and Frankel *et al*.[22] reported a positive association between seropositivity and previous history of abortions No statistically significant association of seropositivity to HSV-2 with respect to history suggestive of other sexually transmitted infections and HIV serology was seen in our study. Similar findings have been reported by Chawla *et al*.[18]

A high frequency of unrecognized and asymptomatic HSV-2 infections was observed in our study; only one out of 15 seropositive cases (6.7%) reported a history of genital herpes, although 42% of these cases complained of vague symptoms like itching discharge, dysuria, discomfort, etc. The increasing prevalence of genital herpes, high frequency of asymptomatic and unrecognized infections, high rate of recurrence, potential for transmission to neonate and lack of a definitive cure have made this disease of great concern. Another aspect of genital herpes is that transmission can occur in long-standing monogamous relationships, a low-risk group for sexually transmitted infections, as the virus may be transmitted to susceptible partner after a long time of sexual contact because infection due to unrecognized reactivation in the infected partner is intermittent. Our findings suggest that HSV-type-specific serotesting could be an efficient strategy to diagnose clinically asymptomatic HSV-2 infections and, therefore, to reduce the risk of HSV-2 and HIV sexual transmission by prophylactic counseling against unprotected intercourse. It may also be a useful adjunct in detecting cases who present with symptoms not directly suggestive of genital herpes. Type-specific serological screening has been recommended to identify women at risk of acquiring genital HSV-2 infection close to term when there is a high (30–50%) risk of neonatal herpes.

However, our study is a hospital-based study, carried out on a small sample of patients and contains no data on men. A large-scale seroepidemological survey may be helpful in assessing the burden of disease and also the utility of type-specific serotesting for HSV-2.
